# Recent Progress Regarding the Molecular Aspects of Insect Gall Formation

**DOI:** 10.3390/ijms22179424

**Published:** 2021-08-30

**Authors:** Seiji Takeda, Tomoko Hirano, Issei Ohshima, Masa H. Sato

**Affiliations:** 1 Graduate School of Life and Environmental Sciences, Kyoto Prefectural University, Shimogamo-Hangi-cho, Sakyo-ku, Kyoto 606-8522, Japan; seijitakeda@kpu.ac.jp (S.T.); thirano@kpu.ac.jp (T.H.); issei@kpu.ac.jp (I.O.); 2 Biotechnology Research Department, Kyoto Prefectural Agriculture Forestry and Fisheries Technology Center, Kitainayazuma Oji 74, Seika, Kyoto 619-0244, Japan; 3 Center for Frontier Natural History, Kyoto Prefectural University, Shimogamo-Hangi-cho, Sakyo-ku, Kyoto 606-8522, Japan

**Keywords:** adaptive significance of insect galls, gall-inducing insects, gall formation mechanism, insect effectors

## Abstract

Galls are characteristic plant structures formed by cell size enlargement and/or cell proliferation induced by parasitic or pathogenic organisms. Insects are a major inducer of galls, and insect galls can occur on plant leaves, stems, floral buds, flowers, fruits, or roots. Many of these exhibit unique shapes, providing shelter and nutrients to insects. To form unique gall structures, gall-inducing insects are believed to secrete certain effector molecules and hijack host developmental programs. However, the molecular mechanisms of insect gall induction and development remain largely unknown due to the difficulties associated with the study of non-model plants in the wild. Recent advances in next-generation sequencing have allowed us to determine the biological processes in non-model organisms, including gall-inducing insects and their host plants. In this review, we first summarize the adaptive significance of galls for insects and plants. Thereafter, we summarize recent progress regarding the molecular aspects of insect gall formation.

## 1. Introduction

Galls are induced on plants by viruses, mycoplasma, bacteria, fungi, nematodes, insects, mites, and other plants. They are defined by an abnormal plant organ development with ectopic cell proliferation and expansion, generating a wide range of gall morphologies [1,2]. Among them, insect-induced galls have attracted the attention of many researchers because of their unique shapes and wide range of variation. The estimated number of gall-inducing insects ranges from 21,000 to 211,000 [3,4]. Furthermore, host plant species span numerous phylogenetic lineages, suggesting that gall-inducing systems have evolved independently during the insects evolution [3,4,5,6].

**Figure 1:**
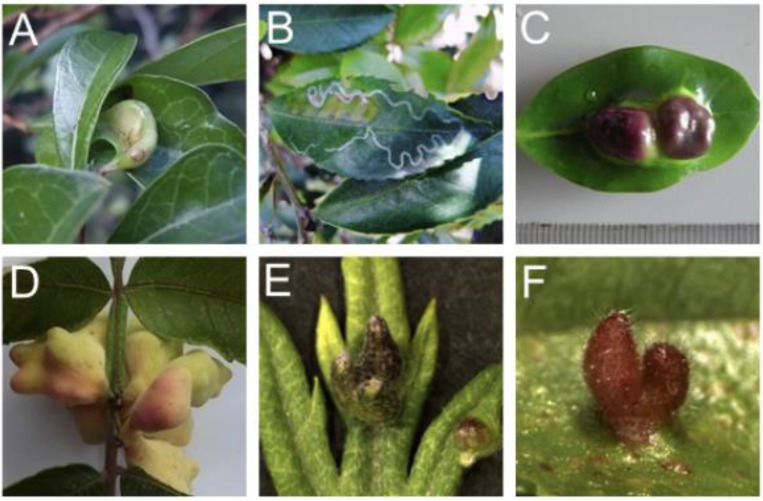
Examples of insect galls generated on leaves. (**A**) *Glochidion obovatum* gall by the micromoth *Caloptilia cecidophora*. (**B**) *Eurya japonica* gall by the micromoth *Borboryctis euryae*. (**C**) *Distylium racemosum* gall by the aphid *Neothoracaphis yanonis*. (**D**) *Rhus javanica* gall by the aphid *Schlechtendalia chinensis*. (**E**) *Artemisia montana* gall by the gall midge *Rhopalomyia yomogicola*. (**F**) *Ulmus parvifolia* gall by *Tetraneura akinire*. Panels (**A**,**D**) are from a previous study [7].

Insect galls can be induced on plant leaves, stems, floral buds, flowers, fruits, or roots, and exhibit unique shapes (Figure 1).

Although galls often resemble fruits or floral buds, their morphologies are generally considerably different from those of plant structures. Therefore, gall-inducing insects appear to hijack the plant developmental system to generate a novel structure in plants. Gall-inducing insects produce stimuli that initiate the development and maintenance of gall tissue. Interestingly, phytohormones, such as indole acetic acid and cytokinins, are detected at higher concentrations in gall-inducing insects than in galls generated on host plants [8,9,10,11,12]. There is also evidence that certain amino acids and proteins are possible signals for gall induction [6]. These results suggest that substances transferred to plants via an insect ovipositor, secreted from larval saliva and/or excrement from larvae, induce the reprogramming of plant cells [5,6,13].

The unique shapes and wide range of variation in galls have attracted the attention of researchers in entomology, botany, and ecology, as well as amateur nature enthusiasts. Each specific insect species generates more or less a fixed gall shape on their respective host plants, indicating that gall-inducing insects activate specific developmental pathways of each host plant and tightly control these pathways. However, the molecular and cellular mechanisms of gall development remain poorly understood, and further studies are needed that focus on a variety of insect and plant taxa.

In this review, we assess insect galls from the perspective of plant molecular biology, with the hope of promoting future molecular plant studies on insect galls. First, we review the evolutionary aspects of the gall-inducing life histories of insects and plants and their adaptive significance. We then briefly review the recent advances made in the molecular aspects of insect gall formation.

## 2. Significance of Morphology and Function of Galls for Insects

**Figure 2:**
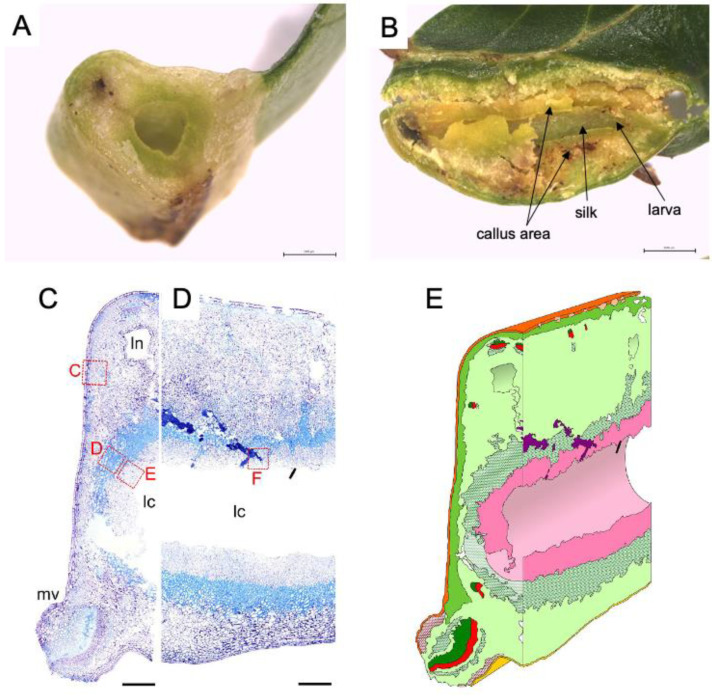
Inside of leaf gall on *Glochidion obovatum*. (**A**) Transverse section. (**B**) Sagittal section showing callus-like cells, silk covering the chamber wall, and larva inside the chamber. (**C**,**D**) Histological transverse (**C**) and sagittal (**D**) sections. Ln: lacuna, lc: larva chamber, mv: midvein. (**E**) 3D representation of gall inside based on histology sections. Dark green: xylem, red: phloem, pink: nutritive tissue, green cross: sclerenchyma, red cross: collenchyma, purple: frass. Bars: A, B, 1 mm; C, D, 0.5 mm. Panels are cited from [7,18].

The shape and cell status of galls have raised three major hypotheses for their adaptive significances of gall-inducing insects: nutrient hypothesis, microenvironment hypothesis, and enemy hypothesis [3,6]. These are profits of galls for insects, rather than host plants. The nutrient hypothesis explains that galls play a role as a nutrient source for insects, and in turn, insects somehow keep regulating plant cell differentiation to provide nutrients, since gall-inducing insects can live within galls for several months. For instance, aphid–induced galls on leaves accumulate a much higher amount of amino acids than intact leaves, suggesting that the galls provide a nitrogen source to gall-inducing insects [14,15]. Photoassimilates accumulate in some aphid-induced galls, suggesting that galls act as a sink organ [16,17]. Structurally, leaf galls of *Glochidion obovatum*, induced by micromoth *Caloptilia cecidophora*, generate several cell layers with different characters surrounding the central chamber, one of which is speculated to be nutritive tissue (Figure 2) because the tissue is almost completely consumed by the pupation of inside larvae [18]. Another type of leaf gall on *Eurya japonica* induced by micromoth *Borboryctis euryae* are much thinner compared to the *G. obovatum* galls and are called mine-galls, but larvae actively induce callus proliferation within the leaves, and feeding on the induced tissues are essential for the pupation [19]. These results strongly support the hypothesis of galls as nutrient source for gall-inducing insects. Interestingly, both *C. cecidophora* and *B. euryae* show mixed feeding habits, where early instars are normal leaf miners, but larvae suddenly begin inducing galls after molting into species specific instars [18,19]. Recent phylogenetic studies clearly show that the two gall-inducing leaf-miner species evolved independently from normal leaf miners [20,21]. The fact that both *C. cecidophora* and *B. euryae* cannot complete their larval period without feeding on gall tissues, coupled with their phylogenetic positions, indicates that these gall-inducing insects lost their ability to survive by feeding only on normal leaf tissues during the course of the evolution of their gall-inducing ability, and that some nutrients obtained from gall tissues are not supplemental but vital for their survival.

The enemy hypothesis explains that the galls protect the residential insects from their enemies and pathogens. Many galls have enclosed environments surrounded by the lignified cell layer for protecting the insects inside of the gall against attack by nonspecialist predators and pathogens [22]. However, the gall structures never provide complete enemy-free space: there are many specialist enemies including fungi, wasps, beetles, moths, and flies, which confer higher mortality [3,6,23]. Nevertheless, the enemy hypothesis is supported for the case of gall-inducing sawflies which are attacked by fewer parasitoid species with lower mortalities than free-living forms [24].

The microenvironment hypothesis states that gall tissues would protect gall-inducing insects from unfavorable abiotic stress conditions such as temperature and humidity [3,25,26]. For instance, when a hole is made on the closed gall of *Distylium racemosum* generated by social aphid *Nipponaphis monzeni*, many soldier nymphs inside the gall gather to the hall and discharge their body fluid to plug the hall, as well as activating plant cell proliferation around the hall [27]. This strongly suggests that the gall-inducing insects actively maintain the microenvironment within the gall. Humidity is suggested to be important for insects, since desiccation can be lethal for their lives [3,6]. There are ambiguous supports for temperature and protection: the temperature does not differ significantly between the inside and outside of galls, and there are many species of both parasitoids and inquilines targeting the gall-inducing insects [3,6,28]. Although the lignified cell layer of galls suggests the function as a shelter from predators, more investigation would be required to confirm this hypothesis.

It is interesting that the outer surface of some galls turns red, due to accumulation of secondary metabolites such as anthocyanins (Figure 1). Red is the contrast color of green and must be remarkable to birds or mammalian animals, a candidate predator of insects. Three hypotheses have been suggested for significance of gall coloring for distinct reasons: (i) representing aposematic or warning coloration to potential enemies of gall-inducing insects, (ii) causing early senescence of the plant tissue for nutrient translocation, and (iii) an adjunct during gall induction caused by cytokinin and sugar [23,29,30]. Another possibility is that, if galls affect plant growth negatively, turning the surface color with accumulation of anthocyanin could be a plant response to remove the galls by predators such birds, but evidence supporting this hypothesis has not been reported thus far. Alternatively, since galls carry molecular features such as flowers and fruits (shown by transcriptome analyses: see below), coloration of galls are one of the characteristics of these reproductive organs. There are several other hypotheses, therefore the coloration of galls would be an interesting phenomenon worth investigating its adaptive significance.

## 3. Benefits of Gall Formation for Plants

Galls do not appear to carry adaptive benefits for plants; on the contrary, they either negatively affect plant growth or have no effect, as observed in many cases. Plants may generate galls to localize parasitic gall-inducing insects in a limited space, allowing plants to inhibit the spread of damage, although evidence supporting this hypothesis is poor [1,3]. Another hypothesis is that plants use excrement from gall-inducing insects by absorbing them from galls. For instance, in closed galls of *Distylium racemosum* and *Syrax japonicus*, honeydew from gall-inducing aphids is absorbed by the galls through the plant vascular system, and this mechanism helps aphids avoid drowning in their secretions within the gall. This suggests the possibility that plants use secretions from gall-inducing insects; however, the sugar concentration of honeydew from these aphids is low and devoid of sucrose [31]. In the *S. chinensis-Rhus chinensis* galling system, the gall and the aphids inside are composed of a metabolic and nutrient exchange hub that benefits not only the aphid but also its host plant. Consequently, host plants provide both shelter and nutrients, and in turn, aphid-derived metabolites compensate host metabolism to a certain extent [32]. A gall-inducing weevil, *Smicronyx madaranus* forms spherical galls on the shoots of an obligate parasitic plant, *Cuscuta campestris*. Parasitic *C. campestris* usually shows low chlorophyll content and has less photosynthetic activity because it usually depends on host plants for its nutrients. However, *S. mandaranes*-induced galls have significantly increased chlorophyll content and photosynthetic activity, suggesting that the gall-inducing weevil enhances the photosynthetic activity to modify the plant developmental programs for producing insect nutrients [33].

The galled shoot of *Silphium integrifolium* formed by the cynipid wasp, *Antistrophus silphii* increases photosynthesis activity, stomatal conductance, and xylem water potential in drought-stress conditions compared with the ungalled shoot, suggesting that the cynipid wasp enhances photosynthesis and water potential not only in gall tissues but also in whole shoots attached to galls [34]. As another example, *Eucalyptus* plants with galls exhibited indirect effects, resulting in tolerance to cold injury, compared to those without galls [35]. Although these examples show the benefits of galls for plants, direct or indirect evidence is required to elucidate the benefits derived from galls in plants.

## 4. Molecular Biology in Host Plants in Insect Gall Formation

### 4.1. Changes in Plant Hormonal Regulation during Gall Development

The common characteristics of complex insect gall structures include the lignified outside cell layer, which has a protective function against natural enemies and the outside environment, and the nutritive tissues of callus-like cells, which contain insect nutrients and vascular tissues to transport nutrients to these tissues [1,6,18,19]. To construct gall structures, many gall-inducing insects are believed to have the ability to secrete certain effectors, including plant hormones, into plant tissues using their mouthparts or ovipositors to induce gall formation in the host plants [5,8,11,36,37]. To induce complex gall structures in plant tissues, gall-inducing insects must control the cellular machinery of their hosts, including development, metabolism, chemistry, and physiology to hijack host gene expression programs in favor of those of the insect.

During the growing process of the gall tissues, a combination of cell division and cell growth occurs by the action of gall-inducing insects. In this process, phytohormones produced by gall-inducing insects have long been hypothesized to play a key role in inducing gall structures to exogenously control phytohormone regulatory pathways in host plants [9,12,38]. However, little was known regarding the gene expression profiles in developing galls because of the difficulty of studying non-model plants in the wild. Recent progress in next-generation sequencing (NGS) has allowed us to determine the structure of biological processes in non-model organisms, including gall-inducing insects and their host plants. Recently, several transcriptome analyses indicated that a significant number of phytohormone-regulatory genes are upregulated in various developing galls.

For instance, a transcriptome analysis of psyllid galls on the Hawaiian *Metrosideros polymorpha* showed that auxin response genes were upregulated in galls [39]. In galls formed on *R. chinensis* and *R. javanica*, the genes involved in the metabolic and signal transduction pathways of auxin and cytokinins were found to be substantially activated [40,41].

Comparison of transcripts in four different galls, suggested that 38 genes are commonly up regulated. GO analysis showed that peptide biosynthesis and metabolism are commonly involved in the four different galls [7]. Among these, AtMYB77 is involved in lateral root formation via auxin signaling [42]. Since Arabidopsis cell regeneration mediates the process of lateral root development [43], the callus generation within the gall may be mediated by AtMYB77 and auxin signaling.

### 4.2. Attempts to Identification of Effector Molecules Involved in the Gall Formation

In addition to the effects of insect-derived phytohormones for inducing gall structures, other effector molecules are believed to be needed for constructing complex insect gall structures [5]. A gall midge, the hessian fly (*Mayetiola destructor*), produces nutritive tissue which acts as a sink tissue with its host plant [44]. The hessian fly was the first gall-inducing insect for which the genome and salivary gland transcriptome and proteome were published [45,46,47]. The genome provided evidence for hundreds of transcripts encoding candidate effectors [47]. Map-based cloning identified four candidate avirulence effectors (vH6, vH9, vH13, and vH14) [47,48,49]. RNA-interference-knockdown of the vH13 gene confirmed that knockdown of that the gene allows larvae to survive on H13-resisitant plants, suggesting that the vH13-encoded protein is an avirulence effector, but not a gall-inducing effector [48].

Gall-inducing effector candidates have been identified from the transcriptome analysis of ovaries and venom glands of two cynipid gall wasps, *Biorhiza pallida* and *Diplolepis rosae*, inducing galls on oak and rose, respectively [50], or the analysis of salivary gland proteome of root-galling grape phylloxera, *Daktulosphaira vitifoliae* [51]. However, there is no direct evidence showing that these effector candidates have gall-inducing activity in their host plants.

Recently, a novel insect secretory protein, designated as BICYCLE, was identified from the gall-inducing aphid *Hormaphis cornu*, which produces distinctive cone galls on the leaves of *Hamamelis virginiana* [52]. *Bicycle* genes were most strongly expressed in the salivary glands of gall-inducing generations. These results suggest that BICYCLE proteins regulate the gall development process in *H. virginiana*. A insect-derived plant regulators, Bruchins identified from both cowpea weevil (*Callosobruchus maculatus* F.) and pea weevil (*Bruchus pisorum* L.) were revealed to show neoplasm formation activity on pods of all of the pea line tested when few amounts of these compounds were applied to intact plants [53]. Thus, several effector candidates have been identified. However, because of the lack of model systems for analyzing gall-inducing insect and host plant interactions, it is not known whether these effector candidates indeed have a gall-inducing function. Future studies are needed to establish a model system for assessing gall induction and development processes at the molecular level.

### 4.3. Changes in the Expression Patterns of Genes Involved in the Biosynthesis of the Metabolic Process during Gall Formation

Tannins are crucial for the protection of host plants and gall-inducing insects from herbivores. Aphid galls on *R. chinensis* accumulate gallotannin, and genes involved in gallotannin biosynthesis [54], gallic acid synthesis [41], and lignin biosynthesis [40] have been identified. In the developing gall of the chestnut gall wasp, *Dryocosmus kuriphilus*, on the Chinese chestnut, *Castanea mollissima*, the expression of genes related to metabolic processes, such as phenylpropanoid biosynthesis, secondary metabolism, and plant-pathogen interactions, was altered compared to that of non-infested leaves [55]. Galls induced on elm leaves by a gall-inducing aphid, *Tetraneura akinire*, were shown to express the genes encoding lignocellulose synthase, suggesting the reinforcement of cell walls to improve resistance to damage by aphids [56].

### 4.4. Regulation of Transcriptional Factors for Reproductive Organ Development

Darwin proposed that the shapes of complex insect galls resemble those of flowers or fruits [57]. Indeed, many remarkable flower- and fruit-like structures have been observed in insect galls, particularly those induced by gall midges, aphids, and cynipids in various host plant species [58], suggesting that the formation of gall tissues is similar to the development of flowers or fruits [59,60]. It has recently been shown that genes involved in the development of flowers and fruits are activated in the leaf galls in *Vitis riparia* induced by the phylloxera, *D. vitifoliae* [60].

Activation of reproductive organ genes has also been reported during the early development of galls on *R. javanica* induced by a gall-inducing aphid, *S. chinensis*. Additionally, class-1 KNOX transcription factors were shown to be overexpressed ectopically in the early developmental gall tissue of *R. javanica*. Because the class-1 KNOX transcription factors are known to lead to the formation of de novo meristematic structures in leaves [61], the results support the hypothesis that gall-inducing insects convert source tissues into meristematic tissues by expressing the class-1 KNOX genes in leaf tissue cells.

This hypothesis was partly supported by comparative transcriptome analysis using four different insect galls generated on leaves: galls on *G. obovatum* induced by the micromoth *C. cecidophora*, on *E. japonica* by the micromoth *B. euryae*, *R. javanica* by the aphid *S. chinensis*, and on *Artemisia montana* by the gall midge *Rhopalomyia yomogicola*. Comparison of these different galls revealed that (i) photosynthetic genes are downregulated in galls, supporting the hypothesis that galls are converted to sink organs rather than source organs on leaves; (ii) developmental, cell cycle, and phytohormone genes are upregulated in galls; and (iii) approximately 40 genes are commonly upregulated in these four galls. In particular, several key regulators of stem cell generation (*CLE44*, *BAM3*, *WOXs*) [62,63] and maintenance floral organ development (*SEPALLATA*, *AGAMOUS*, and *APETALA1*) [64] were commonly upregulated in *A. montana*, *G. obovatum*, and *R. javanica* galls. Intriguingly, these floral organ regulatory transcription factors were not expressed in the *E. japonica* gall tissues. This gall has rather thin layers with a simple pouch-type gall structure compared with the other fruit-type galls, suggesting that these transcription factors do not induce the galls of *E. japonica*. We realize that the comparison of only four species is not sufficient to cover a wide range of variations in gall morphology. However, the accumulation of this type of comparable data will reveal the common mechanisms of gall development and those that generate diversity in galls.

**Figure 3:**
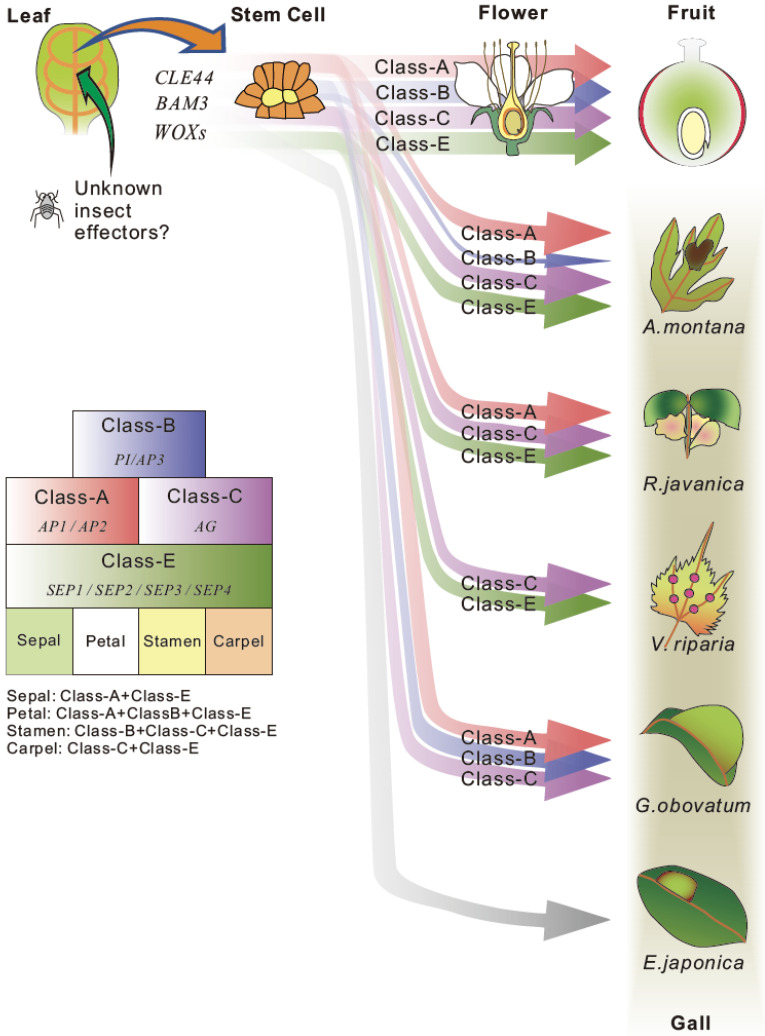
A hypothesis of gall formation processes in various host plants. Common candidate genes for stem cell generation and floral organ development were extracted from transcriptome data of developing galls of *A. montana*, *G. obovatum* [7], *R. javanica* [40] and *V. riparia* [60]. The thickness of arrow indicates the expression level of each gene. The gall formation hypothesis explains the gall formation mechanism that sepal and carpel-based galls are generated by the ectopic over expression of class-A, class-B, and class-E floral homeotic genes on leaves in *A. montana* and *R. javanica*, or carpel-based gall is generated by the expression of class-C and E genes in *V. riparia*. No floral homeotic genes need for generation of pouch-type thin gall of *E. japonica*.

Collectively, we propose following processes that occur during the early stage of gall formation: (i) unknown insect effectors alter the gene expression in host plant organs (e.g., leaves); (ii) source organs (leaves) change their cell fate to meristematic character; (iii) a different combination of floral homeotic genes generate various gall structures (Figure 3).

## 5. Conclusions

Herein, we reviewed the molecular aspects of insect gall induction and development. The ancestral lifestyle of gall-inducing insects varies among taxa, from ectophagous herbivores (foliage feeders, sap-feeders, and pollen-feeders), plant-mining herbivores (leaf-miners and stem-borers), and fungus-feeders to parasitoids. Reports on the origin of leaf miners are scarce. Regarding the adaptive significance of galls for insects, the nutritive hypothesis has received the most experimental support through various direct approaches, including chemical analyses and bioassays. In contrast, enemy and microenvironment hypotheses are only supported by indirect evidence, such as comparisons of predation rates by enemies between taxa with and without a gall-inducing lifestyle and comparisons of the numbers of gall-inducing species between xeric and mesic habitats. Although gall morphologies have received attention in terms of protection from predators and harsh environments, few studies have been conducted on the coloration of galls. The adaptive significance of coloration is an interesting topic worthy of investigation. Although very few studies have been conducted on the plant benefits of galls, one study found that gall-induced plants have increased tolerance. Thus, more studies will be needed to elucidate the generality of this phenomenon. Finally, we note that an increasing number of studies challenge the molecular aspect of gall induction and development, and recent studies have revealed that the formation of gall tissues is similar to the development of flowers or fruits in terms of gene expression patterns. Therefore, studies using a wide range of gall-inducing insects and their host plant taxa would yield more detailed insights into molecular-level insect-plant interactions, which may be divided into subcategories. These studies should provide concise and precise descriptions of the experimental results, their interpretation, and the experimental conclusions drawn.
